# Adherence to exercise after an acute coronary syndrome: a 6-month randomized controlled trial

**DOI:** 10.3389/fphys.2024.1319907

**Published:** 2024-01-26

**Authors:** Essi O. Saarikoski, Elina T. M. Roiha, Antti M. Kiviniemi, Jose Cerdán-De-las-Heras, Juha Perkiömäki, Kari S. Kaikkonen, Mikko P. Tulppo

**Affiliations:** ^1^ Research Unit of Biomedicine and Internal Medicine, Medical Research Center Oulu, Oulu University Hospital and University of Oulu, Oulu, Finland; ^2^ Department of Respiratory Diseases and Allergy, Aarhus University Hospital, Aarhus, Denmark

**Keywords:** coronary artery disease, exercise training, heart rate variability, aerobic training, resistance training

## Abstract

**Introduction:** Exercise training with well-known health benefits is a key element in the self-management of coronary artery disease (CAD). Although current guidelines for patients with CAD recommend daily exercise training, most of the patients do not follow the guidelines. We tested the hypothesis that an exercise training program guided by a novel technology used at home will improve adherence to exercise training.

**Methods:** One to three weeks after percutaneous coronary intervention (PCI), acute coronary syndrome patients (*n* = 50) were randomized into traditional (age 65 ± 8 years) and novel technology-guided (age 60 ± 8 years) exercise rehabilitation groups. The novel technology included a tablet computer with a virtual autonomous physiotherapy agent (VAPA group) for every patient at home; it was used to guide exercise training time, volume, and intensity. Traditional rehabilitation was performed by exercise training prescriptions, phone calls, and diaries (control group). The duration of the rehabilitation program was 6 months for both groups. Exercise capacity and 24-h heart rate variability were measured at baseline and at the end of the program. Adherence to exercise was measured over 6 months as the percentage of realized training.

**Results:** None of the patients dropped out from the VAPA group, while three patients dropped out from the control group. Adherence to exercise was higher in the VAPA group than in the control group for resistance training (141% ± 56% vs. 50% ± 20%, *p* < 0.0001), and there were no differences between the groups for aerobic training (144% ± 45% vs. 119% ± 65%, *p* = 0.22). Exercise capacity increased in both the groups (time *p* < 0.001, time × group interaction p = ns). High-frequency power of R-R intervals (lnHF) increased in the VAPA group but remained unchanged in the control group (natural logarithm of lnHF power from 5.5 ± 0.7 to 5.8 ± 0.9 ms^2^ and from 5.3 ± 0.8 to 5.2 ± 0.7 ms^2^, respectively, time × group interaction *p* = 0.014).

**Conclusion:** Compared with the use of traditional methods, the use of novel technology at home results in better adherence to exercise, particularly in resistance training, in acute coronary syndrome patients. Second, the VAPA group showed improved cardiac vagal regulation, documented by increased vagally mediated R-R interval fluctuation, compared with the traditional training group (ClinicalTrials.gov identifier: NCT03704025).

## Introduction

Physical activity and exercise training with well-known health benefits are key elements in the management of coronary artery disease (CAD) (Balady et al., 2007; Piepoli et al., 2010). Although current guidelines for patients with CAD disease recommend daily physical activity, many patients do not become or remain regularly active (Wofford et al., 2007; Zhao et al., 2008). Almost half of the patients with CAD do not attend recommended rehabilitation programs (Worcester et al., 2004), and among attending patients, the dropout rate is as high as 40%–50% (Sanderson et al., 2003; Sarrafzadegan et al., 2007; Karjalainen et al., 2015). After cardiac rehabilitation, maintenance of an increased level of physical activity is difficult, although it would be important for sustaining the achieved health benefits. One year after cardiac rehabilitation, only approximately 40% of cardiac patients adhered to physical activity recommendations, i.e., three times or >150 min aerobic training and two resistance training sessions weekly (Dolansky et al., 2010; Guiraud et al., 2012; Karjalainen et al., 2015). Most importantly, our recent economic evaluation of an exercise cardiac rehabilitation program conducted partly at home and partly in a rehabilitation center emphasized that exercise cardiac rehabilitation implemented according to current guidelines is less costly and clinically more effective than usual care in acute coronary syndrome patients (Hautala et al., 2016). Taken together, regular exercise training and physical activity according to current guidelines after a cardiac event saves costs and is clinically effective, but patients’ motivation and adherence to exercise programs are very low. Notably, new strategies are needed to motivate cardiac patients for exercise training and physical activity after cardiac events. We hypothesized that an exercise training program guided by novel technology used at home will improve motivation for exercise training and may result in better adherence to exercise training compared to current guidance.

## Methods

### Study population

**FIGURE 1:**
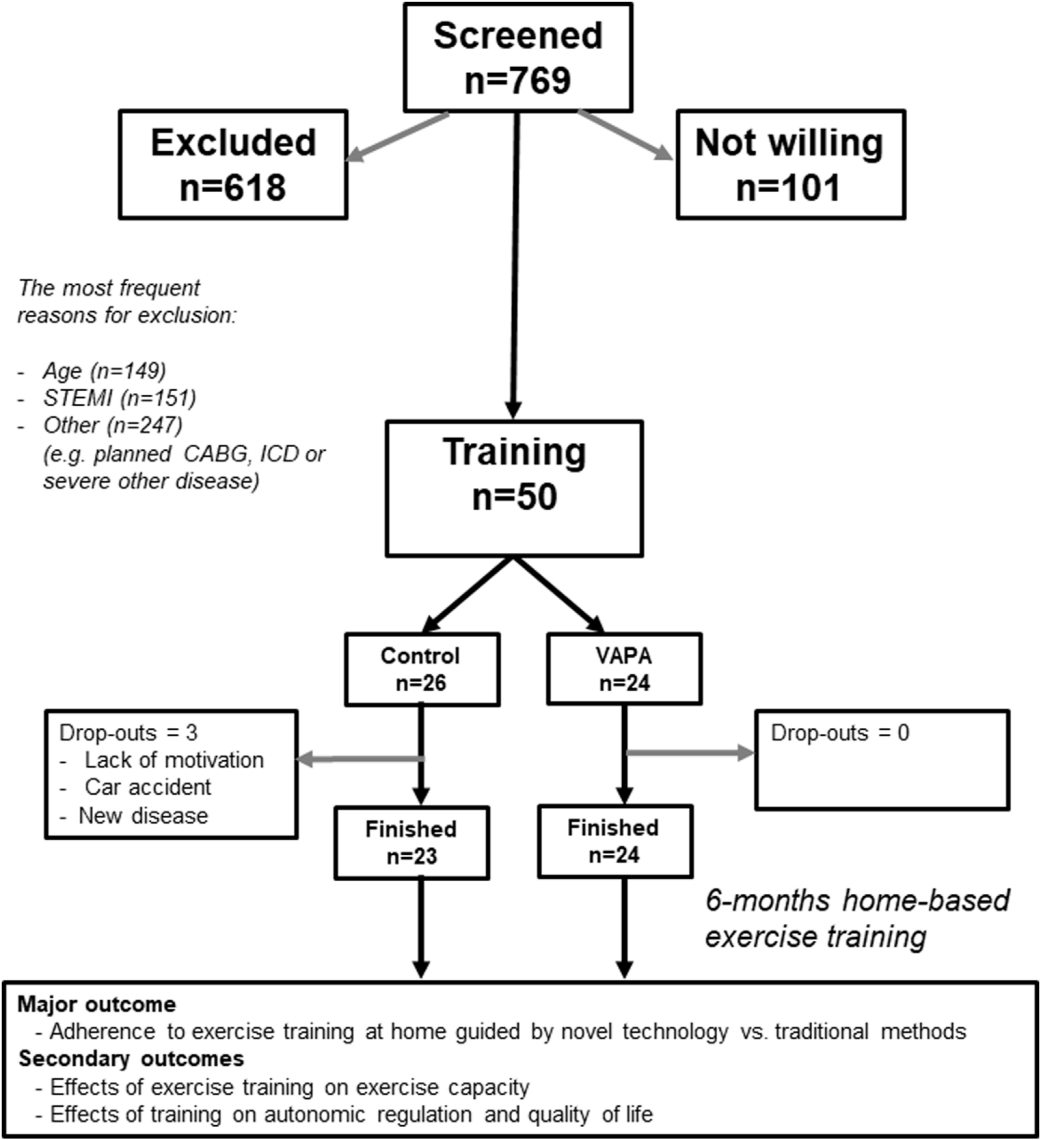
Flow chart of the patient’s requirement.

The subjects (age 63 ± 8; range 47–70 years) of the study were recruited from a consecutive series of patients admitted to Oulu University Hospital due to acute coronary syndrome in December 2017–January 2019 (ClinicalTrials.gov identifier: NCT03704025). All patients underwent coronary angiography and were treated by percutaneous coronary intervention (PCI). A cardiologist determined the applicability of the patients for the study based on their clinical status and the following exclusion criteria: age < 18 or > 70 years, heart failure, Canadian Cardiovascular Society (CCS) class angina pectoris symptoms ≥ 2, implanted or planned cardioverter defibrillator or pacemaker, chronic atrial fibrillation, participation in a competing clinical trial, severe peripheral atherosclerosis, retinopathy or neuropathy, dementia, life expectancy < 2 years due to other serious diseases, or any other reason why the patient was unable or unwilling to provide written informed consent. The patients were informed about the study (description of the study, subject information, and consent document). The study was performed according to the Declaration of Helsinki, the local committee of research ethics of the Northern Ostrobothnia Hospital District approved the protocol, and all the subjects provided written informed consent. The patients’ flow chart is presented in Figure 1. Patients willing to participate were invited to the hospital 1–3 weeks after PCI for an exercise test. Randomization into a virtual autonomous physiotherapy agent group (VAPA, *n* = 25) and control group (*n* = 25) was performed before baseline measurements.

### Study design

The VAPA group had a tablet computer at home with a novel technology to guide exercise training time, volume, and intensity. All the study subjects underwent a thorough clinical examination and health-related questionnaires, performed an exercise test with a bicycle ergometer, and had 24-h ambulatory electrocardiographic (ECG) recordings at baseline and 6 months after exercise training. All the baseline measurements including questionnaires were performed 1–3 weeks after discharge from the hospital (after PCI) (Wijns et al., 2010).

### Exercise training

The duration of the exercise training intervention was 6 months for both groups. Both groups had an exercise training prescription to perform four–five aerobic and two strength training sessions a week according to current guidelines (European Association of Cardiovascular et al., 2010; Fletcher et al., 2013). All the subjects were guided individually on the use of the perceived ratings of exertion (RPE) scale from 6 to 20 to evaluate the average intensity of a single exercise session (Borg, 1982). The patients marked the exercise mode, duration, and mean RPE of each exercise session in the diary or tablet computer. Aerobic training consisted of walking or cycling at the intensity of RPE 10–15 for both groups. Strength training was performed mainly for lower limbs at home by both groups. The intensity of strength training was from low to moderate (RPE 10–15) for both groups.

A written exercise prescription for 1 month was given to the patients in the control group. After the first, third, and fifth months, the subjects were contacted by phone and given new exercise prescriptions for the next months. The VAPA group had tablet computers to motivate and monitor the exercise training program. The tablets had an animated virtual physiotherapist to motivate the patients to do the exercise. The tablets also measured the heart rate (HR) during the exercise. A description of VAPA technology and preliminary results of the VAPA-based exercise training in pulmonary patients have been published recently (Cerdan-de-Las-Heras et al., 2021; Cerdán-de-las-Heras et al., 2022), as have some of the early perceptions and expectations about this novel technology by the test patients (Cerdan de Las Heras et al., 2020).

Realized training load (TRIMP) was calculated from the diaries and by using a computer by calculating training load (RPE × duration of exercise session), as described earlier (Foster, 1998; Hautala et al., 2016). The adherence to exercise training is presented as a percentage of realized TRIPM of planned TRIMP over 6 months separately for aerobic and resistance training.

### Exercise tests

Aerobic capacity (maximal load, W) was measured using a bicycle ergometer test starting from 30 W and increasing by 10 W for women and 15 W for men every 1 min until voluntary exhaustion. ECG was monitored and recorded continuously, and blood pressure was measured at every second load. In the squat test, the subjects stood in front of a chair with their feet shoulder width apart, facing away from it and with hands on hips. The subjects squatted down and lightly touched the chair before standing back up. This was repeated 10 times as fast as possible, and time was measured.

### Heart rate variability

Ambulatory 24-h ECG was measured using a Faros device (sampling frequency 1,000 Hz) to analyze 24-h HR variability (eMotion Faros 360º, Bittium Corporation, Oulu, Finland). HR variability was analyzed using Hearts software (Heart Signal, Kempele, Finland). All R-R intervals were edited by visual inspection based on ECG portions to exclude all the technical artifacts and extrasystole beats, which accounted for < 2% in every subject. Artifacts and ectopic beats were removed and replaced by the local average. However, sequences with ≥10 consecutive beats of noise or ectopic beats were deleted. The measures of R-R interval dynamics were calculated from the entire 24-h period. The R-R intervals were recorded during a non-exercise day before and after the training intervention. At the end of the training intervention, the R-R intervals were recorded after a 48-h non-exercise period (Tulppo et al., 2003). The mean length of R-R intervals and the standard deviation of all R-R intervals (SDNN) were used as time-domain measures of HR variability. An autoregressive model (order 20) was used to estimate the power spectrum densities of R-R interval variability. Very-low-frequency (VLF) power (0.0033–0.04 Hz) was calculated from the entire 24-h segment. Low-frequency (lnLF) power (0.04–0.15 Hz) and high-frequency (lnHF) power (0.15–0.4 Hz) values were calculated from the segments of 512 R-R intervals over the 24-h recording (Malik et al., 1996). The spectral values are expressed as absolute values after a logarithmic transformation, and the LF-to-HF ratio was also calculated as a marker of sympathovagal balance (Pagani et al., 1997).

### Quality of life

The Depression Scale (DEPS) (Salokangas et al., 1995) and Quality of Life questionnaire (15-D) (Sintonen, 2001) were measured for all the subjects at baseline and at the end of the exercise intervention. The DEPS questionnaire contains 10 items, and each item is scored from 0 to 3 in increasing order of severity. A sum of the DEPS score was calculated. In earlier studies, the cut-off point for depression has been ≥ 8 points, showing a sensitivity for depression of 74% to 95% with a specificity for non-depression of 85% to 74% (Poutanen et al., 2007; Poutanen et al., 2010). We have also recently shown that CAD patients with DEPS score ≥ 8 have increased risk for sudden cardiac death (Lahtinen et al., 2018). The 15-D is a generic, standardized, self-administered 15-D instrument intended for measuring health-related quality of life in adults. It can be used both as a profile and as a single index score measure. The 15-D consists of 15 dimensions: mobility, vision, hearing, breathing, sleeping, eating, speech, elimination, usual activities, mental function, discomfort and symptoms, depression, distress, vitality, and sexual activity. Each dimension has five levels of severity, varying from no problem to extreme difficulties (Saarni et al., 2006). The 15-D represents continuous utility scores between 0 (dead) and 1 (full health). The generic minimum important change in 15-D scores is ±0.015 (Alanne et al., 2015).

### Statistics

The normal distribution of different variables was verified using the Kolmogorov–Smirnov goodness-of-fit test or skewness and kurtosis testing. If the distribution was not normal, a logarithmic transformation of the variable was performed prior to statistical analysis (HR variability spectral parameters), or statistical methods suitable for analyzing non-normal distributions were used. Analysis of variance for repeated measurements was used in examining changes in various parameters to detect the significance of the intervention and to make comparisons between groups (time, group, and time × group interaction). HR variability time × group interaction analysis was also adjusted by age and total cholesterol due to the difference in these parameters between groups and well-known effects on HR variability. The Chi-square or Mann–Whitney tests were used to compare averages of class variables and non-parametric variables when appropriate.

## Results

The subjects in the VAPA group were younger and had higher total cholesterol than those in the CONTROL group, and there were no other significant differences in characteristics (Table 1), exercise capacity (Table 2), or autonomic regulation (Table 3) between the study groups at baseline.

**TABLE 1:** Characteristics of patients in different groups at baseline.

Variable	All *n* = 47	VAPA *n* = 24	Control *n* = 23
Age (years)	63 ± 8	60 ± 8	65 ± 8*
Men (%)	35 (75)	18 (75)	17 (74)
Body mass index (kg/m²)	29 ± 5	29 ± 5	29 ± 5
Fat %	28 ± 10	28 ± 10	27 ± 11
Lean body mass (kg)	60.8 ± 9.6	60.1 ± 9.3	62.0 ± 9.9
History of acute myocardial infarction (%)	6 (13)	2 (8)	4 (17)
History of revascularization (%)	12 (26)	7 (29)	5 (22)
Diabetes mellitus (%)	6 (13)	4 (17)	2 (9)
Glycated hemoglobin (mmol/mol)	41 ± 10	41 ± 13	40 ± 8
Total cholesterol (mmol/L)	4.15 ± 1.30	3.77 ± 1.00	4.55 ± 1.47*
HDL cholesterol (mmol/L)	1.17 ± 0.27	1.11 ± 0.25	1.23 ± 0.28
LDL cholesterol (mmol/L)	2.27 ± 1.11	1.96 ± 0.82	2.57 ± 1.30
Triglycerides (mmol/L)	1.64 ± 0.95	1.57 ± 0.93	1.70 ± 1.02
Lifestyle and medication			
Smokers (%)	3 (6)	1 (4)	2 (9)
Alcohol consumers (%)	30 (64)	17 (71)	13 (57)
β-blockers (%)	29 (62)	16 (67)	13 (57)
ACE inhibitors or ATII blockers (%)	40 (85)	19 (79)	21 (91)
Calcium channel blockers (%)	15 (32)	10 (42)	5 (22)
Diuretics (%)	5 (11)	3 (13)	2 (9)
Anticholesterol agents (%)	47 (100)	24 (100)	23 (100)
Quality of life (0–1)	0.94 ± 0.06	0.94 ± 0.06	0.94 ± 0.06
Depression score	2 (0–4)	2 (0–4)	1 (0–3)

Values are expressed as mean (SD), median [1st–3rd quartile (depression score)], or n (% within group). *HDL*, high-density lipoprotein; *LDL*, low-density lipoprotein; *ACE*, angiotensin-converting enzyme; *ATII*, angiotensin receptor II, *p < 0.05, † *p* < 0.01, ‡ *p* < 0.001 between the groups.

**TABLE 2:** Effects of exercise intervention on blood pressure, exercise tests, and quality of life.

Variable		VAPA *n* = 24	Control *n* = 23	Time	Group	Time × group interaction
Weight (kg)	Pre	84.4 ± 13.0	84.9 ± 13.1	ns	ns	ns
	Post	83.8 ± 13.5	85.3 ± 13.5			
Body mass index (kg/m^2^)	Pre	28.8 ± 4.7	29.1 ± 4.7	ns	ns	ns
	Post	28.6 ± 4.7	29.3 ± 4.9			
Body fat %	Pre	28.1 ± 10.2	27.8 ± 11.6	*p* < 0.05	ns	ns
	Post	27.4 ± 10.2	23.8 ± 9.4			
Systolic blood pressure (mmHg)	Pre	132 ± 18	135 ± 18	ns	ns	ns
	Post	133 ± 20	137 ± 16			
Diastolic blood pressure (mmHg)	Pre	84 ± 11	83 ± 11	ns	ns	ns
	Post	82 ± 12	84 ± 9			
Exercise capacity, watts	Pre	155 ± 46	140 ± 32	*p* < 0.001	ns	ns
	Post	167 ± 51	147 ± 31			
Exercise capacity, METS	Pre	7.44 ± 2.11	6.75 ± 1.47	*p* < 0.001	ns	ns
	Post	7.90 ± 2.17	7.04 ± 1.65			
Maximal heart rate (bpm)	Pre	142 ± 17	136 ± 17	*p* < 0.05	ns	ns
	Post	145 ± 16	140 ± 19			
Time for 10 squats (sec)	Pre	10.8 ± 2.6	11.3 ± 3.1	*p* < 0.001	ns	ns
	Post	9.5 ± 1.7	10.3 ± 3.1			
Quality of life (0–1)	Pre	0.939 ± 0.07	0.944 ± 0.05	ns	ns	ns
	Post	0.947 ± 0.07	0.934 ± 0.05			
Depression score (0–30)	Pre	2 (0–4)	1 (0–3)	ns	ns	ns
	Post	1 (0–2)	2 (0–4)			

Values are mean (SD) or median [1st–3rd quartile (depression score)].

**TABLE 3:** Effects of exercise intervention on 24-h heart rate variability.

Variable		VAPA *n* = 23	Control *n* = 23	Time	Group	Time × group interaction	Time × group interaction adjusted
Heart rate (bpm)	Pre	67 ± 8	68 ± 8	ns	ns	ns	
	Post	67 ± 8	69 ± 7				
SDNN (ms)	Pre	154 ± 33	151 ± 31	ns	ns	ns	
	Post	159 ± 42	151 ± 33				
VLF power (ln ms^2^)	Pre	7.54 ± 0.55	7.30 ± 0.42	ns	ns	ns	
	Post	7.55 ± 0.56	7.21 ± 0.45				
LF power (ln ms^2^)	Pre	6.43 ± 0.70	6.19 ± 0.65	ns	ns	ns	
	Post	6.53 ± 0.76	6.14 ± 0.75				
HF power (ln ms^2^)	Pre	5.49 ± 0.68	5.33 ± 0.76	ns	ns	*p* = 0.014	*p* = 0.005
	Post	5.76 ± 0.85	5.23 ± 0.74				
LF/HF ratio	Pre	2.88 ± 1.26	3.04 ± 1.85	ns	ns	*p* = 0.038	*p* = 0.047
	Post	2.48 ± 1.21	3.27 ± 2.16				

Values are expressed as mean (SD), *SDNN*, standard deviation of all R-R intervals; *VLF*, very-low-frequency power; *LF*, low-frequency power; *HF*, high-frequency power. Time × group interaction analysis adjusted by age and total cholesterol. The LF/HF ratio is presented in absolute unit, and statistical analysis is performed after logarithmic transformation.

### Realized exercise training

**FIGURE 2:**
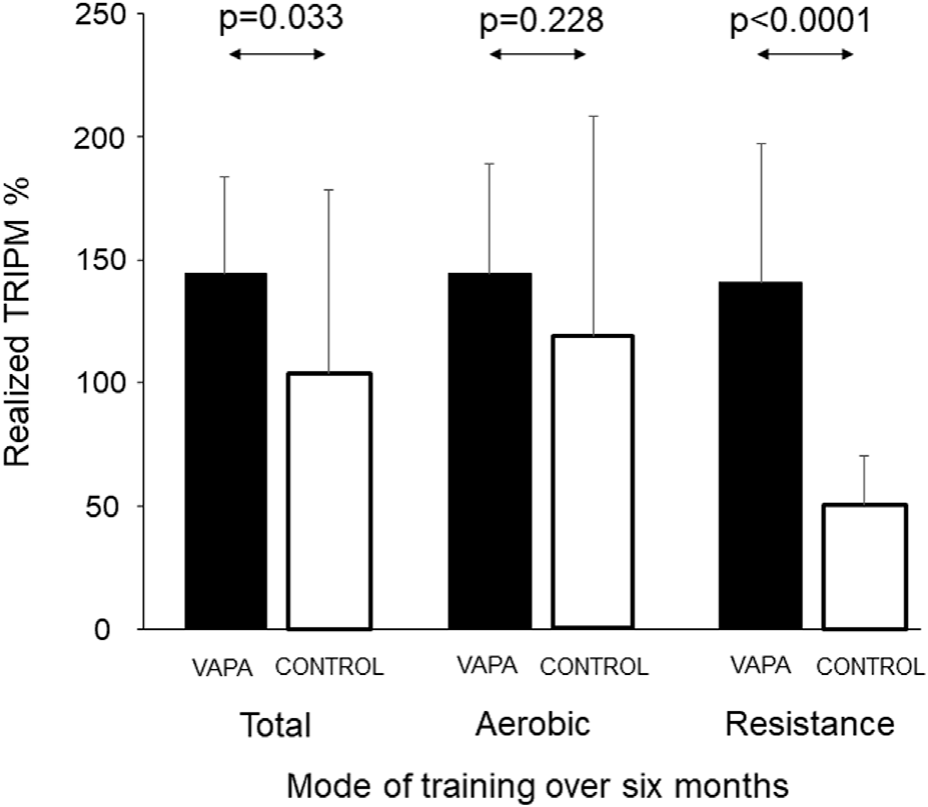
Realized exercise training TRIMP % (exercise time × RPE) as the percentage of planned exercise training analyzed over a 6-month training period separately for aerobic and resistance training.

The average number of aerobic exercise training sessions was 3.4 ± 1.2 vs. 3.4 ± 0.8 per week (*n* = ns) and that of strength training sessions was 0.9 ± 0.3 vs. 1.2 ± 0.4 (*p* < 0.05) per week for CONTROL and VAPA groups, respectively. The adherence to exercise training over 6 months of training is shown in Figure 2. The VAPA group had significantly higher realized TRIMP analyzed particularly over resistance training.

### Effects of exercise training on exercise capacity and autonomic regulation

The effects of training on exercise capacity are shown in Table 2. Maximal aerobic exercise capacity and muscle power increased similarly in both the groups. The lnHF power of the R-R interval, a marker of vagal modulation of the heart, increased in the VAPA group compared to the CONTROL group in multivariate analysis (time × group interaction *p* = 0.014) and after adjustment by age and total cholesterol (time × group interaction *p* = 0.005) (Table 3). Similarly, the LF/HF ratio, a marker of sympathovagal balance, changed toward vagal dominance in the VAPA group and toward sympathetic dominance in the control group (time × group interaction *p* = 0.047 after adjustment) after 6 months’ exercise rehabilitation (Table 3).

### The change in lnHF power and realized training

The change in lnHF power was moderately associated with the average number of resistance training sessions per week (*r* = 0.40, *p* = 0.006) and with realized resistance training TRIMP % (*r* = 0.40, *p* = 0.006) analyzed over all subjects. On the contrary, the change in lnHF power did not correlate linearly with the average number of aerobic training sessions per week (*r* = 0.15, *p* = 0.29) nor with realized aerobic training TRIMP % (*r* = 0.08, *p* = 0.60). There was no significant correlation between the change in lnHF power and other variables at baseline or after training.

### Gender differences

**FIGURE 3:**
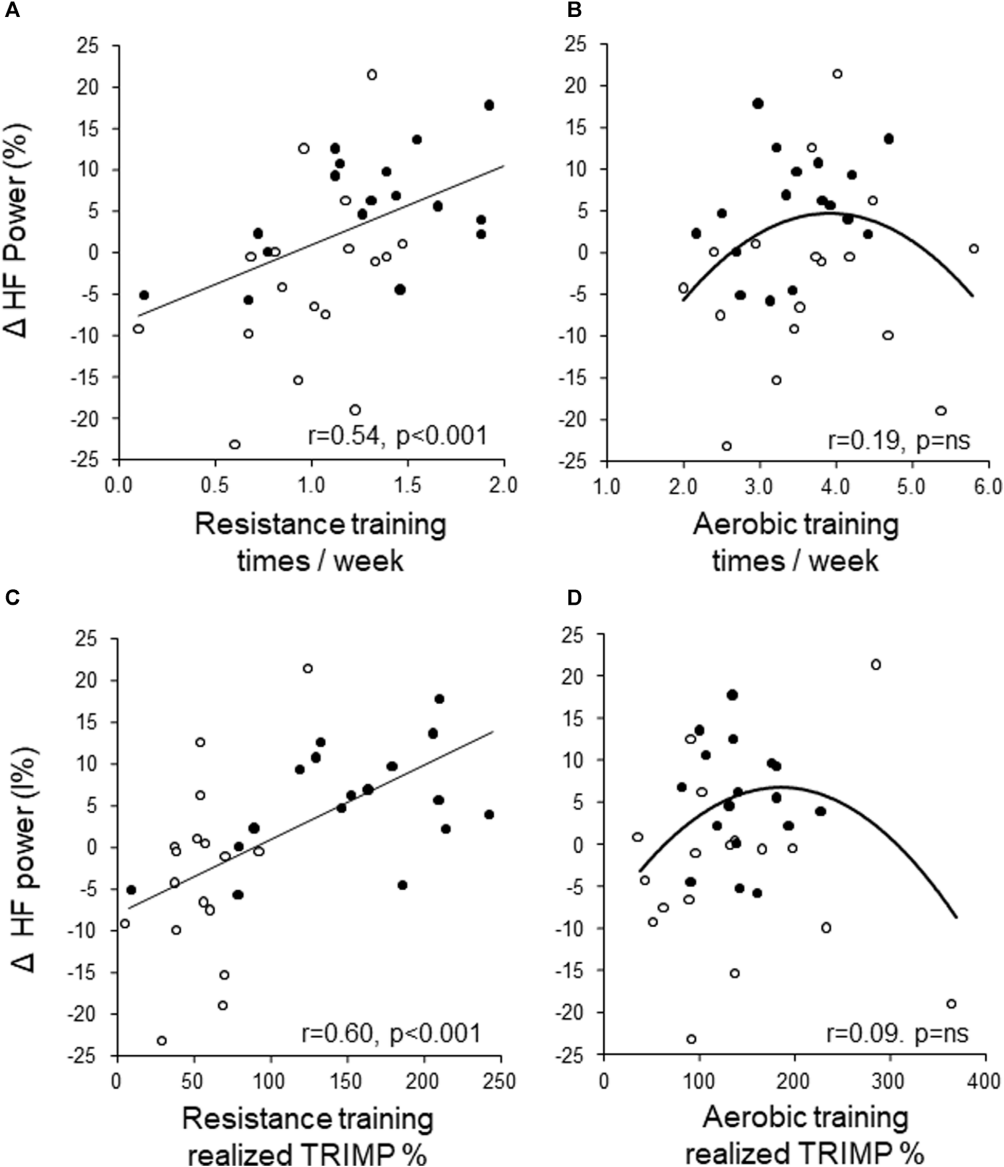
Association between the change in lnHF power and the number of resistance training sessions per week **(A)**, the number of aerobic training sessions per week **(B)**, resistance training TRIPM % **(C)**, and aerobic training TRIMP % **(D)** for male patients, separately for the VAPA (•) and control (ο) groups. Correlations are analyzed over all subjects.

The association between the change in lnHF power and training was analyzed separately for men and women. The association was moderate between the change in lnHF power and the number of resistance training sessions per week (*r* = 0.52, *p* < 0.001) and between realized resistance training TRIMP % (*r* = 0.60, *p* < 0.001) for men. Male subjects were also categorized according to the change in lnHF power as increased lnHF power or decreased/no change groups. Resistance training sessions per week were 1.4 ± 0.3 vs. 0.8 ± 0.4 (*p* < 0.001) and realized resistance training TRIMP % 140 ± 61 vs. 59 ± 42 (*p* < 0.001) for lnHF power increased and decreased/no change groups, respectively. Corresponding values for aerobic training were 3.7 ± 0.8 vs. 3.3 ± 0.8 (p = ns) sessions per week and 137 ± 80 vs. 141 ± 58 (p = ns) realized aerobic training TRIMP % for lnHF power increased and decreased/no change groups, respectively. The association between the change in lnHF power and realized training for male patients, separately for the VAPA and control groups, is shown in Figure 3. There was no association between the change in lnHF power and training in female subjects.

## Discussion

The use of novel technology involving visualized training instruction at home results in superior adherence to exercise rehabilitation compared with traditional methods by exercise prescriptions and phone calls in acute coronary syndrome patients. This was particularly obvious in adherence to resistance training at home, which could be more motivating than the following written programs. Second, cardiac vagal modulation, documented by lnHF power of R-R intervals over 24 h, increased only in the exercise training group guided by novel technology. lnHF power of R-R intervals is a well-known indicator of cardiovascular health. Our finding emphasizes the importance to follow up an appropriate dose and mode of training, both aerobic and resistance exercises, according to current guidelines over weeks and months to improve cardiac autonomic regulation and health.

### New technologies in cardiac rehabilitation

In recent small-scale studies, VAPA technology has proven usable and functional after myocardial infarction and idiopathic pulmonary fibrosis in patients who cannot or are not willing to attend a group rehabilitation program (Cerdan de Las Heras et al., 2020; Cerdan-de-Las-Heras et al., 2021; Cerdán-de-las-Heras et al., 2022). These new technologies seem to increase the motivation and commitment for adopting a more active lifestyle. This might be due to the possibility of following the progression and achievements of rehabilitation on a visual platform, such as a mobile application, a health watch, or a web-based platform, which shows parameters like daily activity or heart rate (Cerdan de Las Heras et al., 2020; Cerdan-de-Las-Heras et al., 2021; Ding et al., 2021). In VAPA-based rehabilitation, the increase in adherence to the exercise program might also be partially explained by the animated character that participates in the exercise with the patient and shows how the movements are done correctly. This is highly likely since the VAPA groups showed better adherence to resistance training, which was guided by visualized animation. This kind of visualized support could be a highly potential method to improve resistance training via computers or cell phones in various other patient groups where muscle strength is a vital parameter for better health, such as older people and type 2 diabetes patients.

### Quality of life

In our study, we were not able to show a significant increase in the quality of life, which is in line with the findings in previous studies (Cerdan-de-Las-Heras et al., 2021; Ding et al., 2021; Ramachandran et al., 2021; Cerdán-de-las-Heras et al., 2022). The average baseline value of the 15-D index score was very high, which may indicate that acute care and medication were well-accepted, and our patients were asymptomatic after PCI intervention at the baseline. Second, the finding may be due to our relatively small study population, the subjective assessment of quality of life by questionnaires, and the follow-up of just 6 months, which is a relatively short time to observe the long-term effects of rehabilitation and/or lifestyle changes. Studies with larger populations and longer follow-ups are still needed. Similarly, there were no significant changes in the depression score, and the depression score was also already rather low at the baseline (after PCI) compared to our consecutive acute cardiac patients’ population study (Lahtinen et al., 2018). Finally, the number of patients who declined to participate was rather high in the present study, and it is highly possible that patients with even a mild depression or symptoms after PCI are not willing to participate in exercise rehabilitation such as the present study.

### Cardiac autonomic regulation

After the 6-month training period, the vagally mediated lnHF power of R-R intervals increased in the VAPA group but did not change or even tend to decrease in the control group. A large body of data show that enhanced vagal influence on HR is generally antiarrhythmic, while increased sympathetic influence is generally pro-arrhythmic (Eckberg et al., 1971; La Rovere et al., 1998; Billman, 2002; Kiviniemi et al., 2007). Increased lnHF power of R-R intervals and decreased LF/HF ratio, as evidence of enhanced cardiac vagal modulation, only after the VAPA program in the present study emphasize the importance of regular exercise training and adherence to current guidelines in aerobic and resistance training. The association between the change in vagally mediated lnHF-power of R-R intervals and the number of resistance sessions is novel and physiological mechanisms only speculative. Successful resistance training is known to result in positive responses on circulating hormones such as testosterone and growth hormone. The potential changes in the hormonal profile due to the resistance training, particularly in male patients, may result in altered cardiac autonomic regulation.

### Limitations

The relatively small population, particularly female patients, is a limitation of this study. However, the results concerning adherence to resistance training were clear. Second, our study population was rather young and from the “healthy end” of acute coronary syndrome patients, which is why older patients and patients with more severe CAD should be included in further studies.

## Conclusion

The use of novel technology at home results in better adherence to exercise, particularly in resistance training, compared with traditional methods in acute coronary syndrome patients. Second, compared with the traditional training group, VAPA results in improved cardiac vagal regulation documented by increased vagally mediated R-R interval fluctuation and decreased LF/HF ratio.

## Data Availability

The datasets presented in this article are not readily available; these datasets will be used in future scientific articles. Requests to access the datasets should be directed to mikko.tulppo@oulu.fi.
